# A Rare Case of Isolated Unilateral Atresia of Pulmonary Artery in an Elderly Female

**DOI:** 10.7759/cureus.5869

**Published:** 2019-10-09

**Authors:** Qurat Ul Ain, Omair A Khan, Mahnoor Sherazi, Mirza Faris Ali Baig, Fakeha Siddiqui

**Affiliations:** 1 Internal Medicine, Medstar Health, Baltimore, USA; 2 Internal Medicine, Fauji Foundation Hospital, Rawalpindi, PAK; 3 Internal Medicine, University of Pittsburgh Medical Center McKeesport, Pittsburgh, USA; 4 Internal Medicine, Dow University of Health Sciences, Karachi, PAK

**Keywords:** isolated atresia of pulmonary artery, congenital disorder, cardiothoracic

## Abstract

Isolated unilateral agenesis/atresia of pulmonary artery (IUAPA) is a rare congenital disorder, an uncommon variant of unilateral agenesis of pulmonary artery (UAPA). Patients with IUAPA may remain asymptomatic and undiagnosed till late adulthood as they present with vague symptoms which may be overlooked. We report a case of IUAPA of right pulmonary artery in an elderly female who presented with complaints of productive cough and exertional dyspnea. Due to the formation of extensive collaterals, her lung parenchyma was preserved.

## Introduction

Unilateral agenesis/atresia of the pulmonary artery (UAPA) is a rare congenital disorder that commonly presents with a cardiovascular disorder [1]. Occasionally, it may occur as an isolated finding, isolated unilateral agenesis of pulmonary artery (IUAPA). In such cases, the patient may present with complaints of cough, exertional dyspnea, recurrent pulmonary infections, pulmonary hemorrhage and pulmonary hypertension, or they remain asymptomatic which is why a correct estimation of its prevalence is hard to make.

Radiological investigation can help in diagnosing this condition. Chest X-ray may show an absent hilar shadow, the affected lung may be shrunken and the mediastinal structures may be shifted to the affected side [2]. Chest computed tomography is also helpful in making a correct diagnosis [3].

Pulmonary angiography is the gold standard to diagnose this rare condition. However, many cases of IUAPA remain undiagnosed as there is no characteristic finding of this condition which may point the physician towards this diagnosis. Keeping this in mind, we report a case of a 69-year-old female with a known history of diabetes mellitus and hypertension, who presented with symptoms of productive cough and exertional dyspnea.

## Case presentation

A 69-year-old female with a past medical history of hypertension, type 2 diabetes mellitus and recent pneumonia presented with complaints of productive cough for two weeks and exertional dyspnea for one day. She confirmed progressively worsening cough, productive of whitish sputum, for the last two weeks. She reported shortness of breath on mild exertion for one day and has also noticed some leg swelling. She denied any fever, chills, night sweats, hemoptysis, weight loss, chest pain or palpitations. She was on metformin 1000 mg twice daily for her diabetes and amlodipine 10 mg daily for hypertension. She denied alcohol abuse, smoking or illicit drug use. There was no relevant family history for any cardiovascular incident.

On presentation at the hospital, initial vital signs were a blood pressure of 144/68 mm Hg, a heart rate of 113 beats per minute, an oxygen saturation of 89% on room air, a respiratory rate of 18 breaths per minute and a temperature of 37.1 degrees Celsius. She was in no apparent acute distress. On lung examination, there were bibasilar crackles and end-expiratory wheezing. There were no carotid bruits or jugular venous distension. The patient was tachycardic; there was no rub or gallop. +1 bilateral peripheral edema up to knees was seen. Pulses +2 equally bilateral in upper and lower extremities were noted.

Her lab investigations were carried out and are shown in Table 1.

**Table 1:** Lab investigations

Investigations	Results (Normal Values)
Sodium level	136 (136-145 mmol/l)
Potassium level	4.3 (3.5-5 mmol/l)
Chloride level	94 (98-107 mmol/l)
Bicarbonate level	25 (22-26 mmol/l)
Blood urea nitrogen	14 (8-23 mg/dl)
Serum creatinine	0.68 (0.7-1.2 mg/dl)
Blood glucose	203 (70-110 mg/dl)
White blood cell count	10 (5-10 cells/mcl)
Hemoglobin	11.3 (12-16 g/dl)
Hematocrit	34.2 (36-44 vol %)
Platelet count	203 (100-400 mm^3^)
Troponin I	<0.015 (<0.07 ng/ml)
N terminal-pro B-type natriuretic peptide	959 (<125 pg/ml)

**Figure 1:**
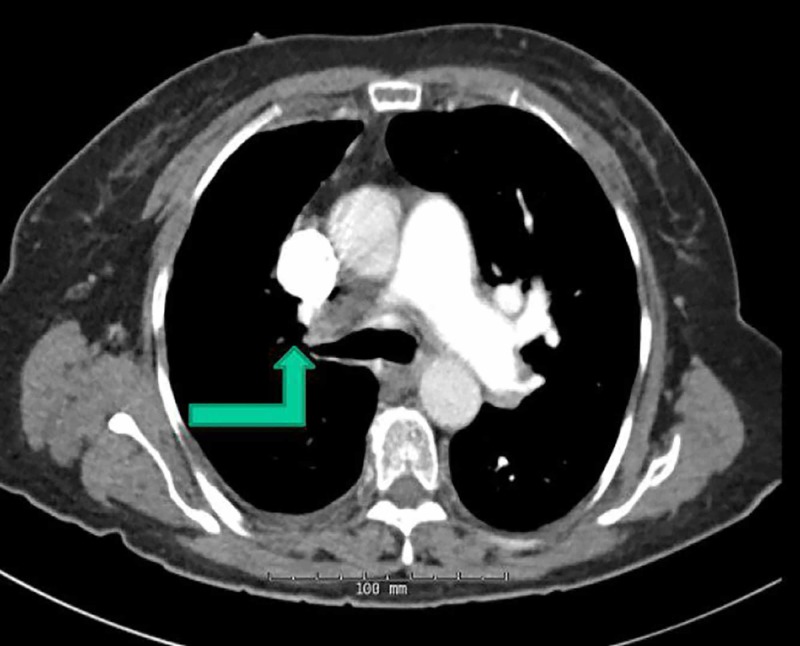
CT angiography showing significant atresia of right pulmonary artery (green arrow)

Several imaging studies were carried out. Chest X-ray showed some alveolar infiltrates consistent with early-onset pneumonia. A bilateral lower extremity duplex was also carried out which excluded deep vein thrombosis. A ventilation/perfusion (V/Q) lung scan showed no significant perfusion to the right lung. CT angiography (CTA) was done to confirm the diagnosis which showed diffusely atretic right pulmonary vasculature indicating pulmonary atresia (Figure 1). On CTA, the left lung was also found to be larger than the right, and a borderline dilated main pulmonary artery in the setting of pulmonary hypertension was observed. Trans-esophageal echocardiography revealed right and left atrial enlargement as well as probable right ventricular enlargement and confirmed severely elevated pulmonary arterial pressure. Ejection fraction was found to be 60%.

The patient was treated with ceftriaxone and azithromycin for community-acquired pneumonia. She was diagnosed with isolated unilateral atresia of right pulmonary artery. The patient was discharged and referred to a pulmonary hypertension clinic and a cardiothoracic surgeon for further management. 

## Discussion

UAPA was first described in 1868 by Frantzel O. Angeborener, and later it was proven angiographically by Madoff and his colleagues in 1953 [4]. It has been estimated that the prevalence of this unique disorder is about one in 200,000 [5]. Involution of the proximal sixth aortic arch of the affected side is the main embryological deficit, which leads to an absence of the proximal pulmonary artery. It has also been perceived that most of the cases affect the right lung and are opposite to the aortic arch [6]. Although most cases of unilateral agenesis of the pulmonary artery are correlated with cardiac abnormalities such as tetralogy of Fallot or septal defects [1], isolated cases are also reported which may present with vague unexplained symptoms. Presenting symptoms in adult patients can be variable such as exertional dyspnea, limited exercise intolerance, hemoptysis, recurrent pulmonary infections and pulmonary hypertension, which is by far the most common presentation among adult population [7]. It has been proposed that alveolar hypocapnia can cause bronchoconstriction, while impaired mucociliary clearance and diminished delivery of inflammatory cells may contribute to the high incidence of respiratory infections [5]. Blood flow to the affected lung is supplied by collaterals arising from bronchial, subclavian, subdiaphragmatic and intercostal arteries; therefore, patients with UAPA may present with hemoptysis [8]. One of the reasons for UAPA remaining undiagnosed for a considerable time is that symptoms are not always well defined, leading to a significant delay (up to >30 years) between the onset of symptoms and the final diagnosis.

A chest radiograph is usually performed as an early diagnostic test. This may show an absent hilar shadow, asymmetric lung fields with a reduced lung volume and a shift of the mediastinal structures to the affected side [9]. Compensatory hyperinflation or a plethoric lung field, due to increased pulmonary blood flow, may be seen on the opposite side [2]. These findings may be subtle or absent as in our case. The diagnosis can be confirmed with noninvasive tests such as CT scan and MRI [10]. A V/Q lung scan can also be performed which will show normal ventilation but no perfusion on the affected side [9]. Pulmonary angiography remains the gold standard for diagnosis but it is not usually performed as non-invasive imaging can help to make a definite diagnosis [10]. Right heart catheterization should be done to assess pulmonary hemodynamics. The presence of hilar arteries can be demonstrated by pulmonary venous wedge angiography [2].

The mortality rate remains 7%-8%, the main culprits being severe pulmonary hypertension and pulmonary hemorrhage [11]. Patients require close follow-up of pulmonary hemodynamics as well as continued medical management for pulmonary hypertension which includes calcium channel blockers, endothelin receptor antagonists and intravenous prostacyclin [2,10]. Selective embolization of the collaterals may be done to control massive hemoptysis [12]. Pneumectomy or lobectomy may be done to help patients with recurrent pulmonary infection [7]. Cardiothoracic transplant is recommended in patients with pulmonary hypertension who have pre-existing congenital heart defect with UAPA [13].

## Conclusions

Clinicians should be aware of the possibility of undiagnosed cases of IUAPA. The diagnosis of IUAPA is challenging due to subtle symptoms. Characteristic chest radiographic changes may be absent as in our case. Pulmonary angiography remains the gold standard for diagnosis. Although there are few reported cases in the literature, the compatibility of such a congenital anomaly with life with minimal to no symptoms continues to be a less recognized phenomenon, which should be explored in patients with recurrent pulmonary infections, hemoptysis or pulmonary hypertension.
